# Overcoming Challenges in Plant Biomechanics: Methodological Innovations and Technological Integration

**DOI:** 10.1002/advs.202415606

**Published:** 2025-01-31

**Authors:** Guanmin Huang, Yuankun Li, Ying Zhang, Weiliang Wen, Chunjiang Zhao, Xinyu Guo

**Affiliations:** ^1^ Information Technology Research Center Beijing Academy of Agriculture and Forestry Sciences Beijing Key Laboratory of Digital Plant China National Engineering Research Center for Information Technology in Agriculture Beijing 100097 China

**Keywords:** future perspectives, interdisciplinary research, mechanical properties, plant biomechanics, research challenges

## Abstract

Plant biomechanics, an emerging interdisciplinary field, plays an irreplaceable role in revealing the structure‐function relationships in plant life processes. This field integrates classical mechanical theories with modern biological methods, providing novel perspectives for plant phenotype research and offering significant theoretical guidance for crop breeding, cultivation management, and ecological protection. This review comprehensively examines existing research from three dimensions: research perspectives, methodologies, and content. Using maize lodging as a case study, key scientific questions, research methods, and modeling strategies are analyzed across scales from molecular to population levels. Furthermore, this paper identifies the main challenges in plant biomechanics research, particularly in methodology development, theoretical framework refinement, model simulation, and 3D modeling. Finally, innovative directions and application prospects are explored for integrating plant biomechanics with artificial intelligence technology, multi‐scale modeling, genetic improvement, and biomimetics. These research advances will pave new paths for theoretical innovation and practical applications in plant biomechanics.

## Graphical abstract

This review systematically examines plant biomechanics research, focusing on maize lodging as a case study to explore structure‐function relationships across multiple scales. The paper analyzes research perspectives, methodologies, and challenges, while highlighting future directions in artificial intelligence integration, multi‐scale modeling, and biomimetics, providing valuable insights for crop improvement and ecological protection.

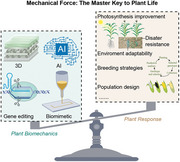

## 1 Introduction

Biomechanics in the field of animal research has made significant progress, accumulating a wealth of theoretical and practical achievements.^[^ ^1^ ^]^ Research in animal biomechanics encompasses various branches such as kinematics, material mechanics, and fluid mechanics. It delves deeply into the mechanical mechanisms behind phenomena like bird flight, fish swimming, skeletal muscle structure, and blood circulation.^[^ ^2^ ^]^ In particular, human biomechanics has achieved remarkable advances in biomedical applications, from orthopedics and rehabilitation to tissue engineering and surgical techniques.^[^ ^3^ ^]^

**Figure 1:**
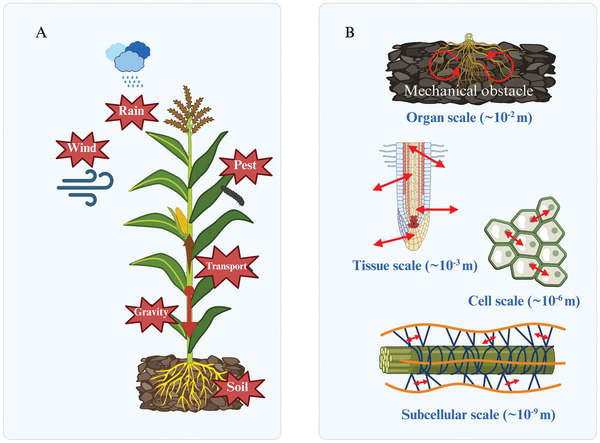
Environmental factors affecting plant growth mechanics and multi‐scale mechanical responses in plants. A. Effects of environmental mechanical factors on plants, including wind, rain, gravity, soil resistance, pest damage, and mechanical stress during transport; B. Multi‐scale mechanical responses in plants, showing force transmission from organ to subcellular level. At the organ scale (≈10^−2^ m), curved arrows indicate bending deformation; at the tissue scale (≈10^−3^ m), arrows represent combined shear and tensile forces; at the cellular scale (≈10^−6^ m), smaller arrows demonstrate intercellular force transmission; and at the subcellular scale (≈10^−9^ m), the smallest bidirectional arrows illustrate molecular interactions between cell wall polymers. The decreasing arrow sizes reflect the hierarchical nature of force transmission through different organizational levels.

These studies have unveiled the mysteries of animal life activities and provided a solid theoretical foundation for fields like biomimetic engineering and medical innovations.^[^ ^4^ ^]^ In contrast, plant biomechanics, as an emerging interdisciplinary field, has started relatively late and developed more slowly.^[^ ^5^ ^]^ Current research primarily focuses on the mechanical properties of plant cell walls and the morphological construction mechanisms of plant tissues.^[^ ^6^ ^]^ Biomechanics, as an objectively existing physical entity, significantly impacts plant growth and development in two main dimensions: environmental and physiological. In the environmental dimension, external mechanical pressures such as wind, gravity, and soil resistance influence plant shape, structure, and growth. These pressures drive plants to optimize their morphology to adapt to their environment, thereby affecting their survival and reproduction.^[^ ^7^ ^]^ In the physiological dimension, the interplay between turgor pressure and cell wall properties drives morphological alterations through three key parameters – growth rate, anisotropy, and direction. Cell division and tissue topology further influence local mechanical signals.^[^ ^8^ ^]^ Through mechanotransduction pathways, these mechanical signals not only regulate plant growth and development processes, but also contribute to essential functions including defense responses, structural reinforcement, stress adaptation and mechanosensing (**Figure** **1**).

Given the importance of plant biomechanical research, a systematic review of developments in this field is crucial. Taking maize as an example, this review systematically summarizes the research content, methodologies, and perspectives in plant biomechanics from molecular to population scales. Research shows that current plant biomechanics studies still face numerous challenges in theoretical frameworks, methodologies, and 3D simulation modeling. Looking ahead, breakthroughs in artificial intelligence technology, multi‐scale modeling, genetic improvement, and biomimetics will provide new pathways and applications to address these challenges. This paper aims to provide reference for theoretical research and practical applications in this field by systematically reviewing advances in plant biomechanics, promote methodological innovation and technological breakthroughs, while offering theoretical guidance for crop adaptability improvement and sustainable agricultural development.

## 2 Literature Review

To comprehensively evaluate the development, current status, and future trends of biomechanical research in plant science, we utilized the Web of Science database from Clarivate Analytics (USA) to retrieve relevant literature published between 2002 and 2023. During the retrieval process, we employed two categories of keywords: general terms such as “Plant Mechanics” and “Plant Biomechanics,” and specific mechanical parameters including force (stress, pressure), deformation (strain, bending), and material properties (viscosity, elasticity). These keywords were combined with structural terms across different scales (from subcellular to ecosystem level) and refined within the Plant Science field. After excluding non‐journal literature such as conference papers, a total of 11375 articles were included. Subsequently, we manually screened the literature based on titles, abstracts, and keywords, ultimately selecting 2881 articles for in‐depth analysis. The analysis covered multiple dimensions, including “Publication year,” “Country,” “Continent,” “Mechanical phenomena,” “Research scale,” and “Scientific questions.” (Table S1, Supporting Information).

**Figure 2:**
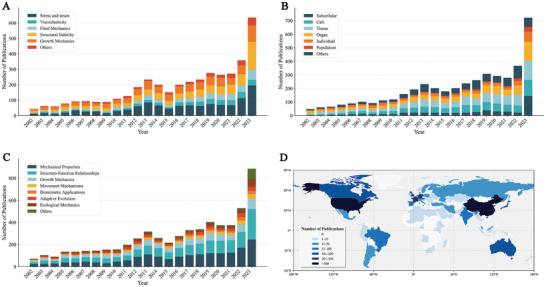
Spatiotemporal Distribution of Publications in Plant Biomechanics Research (2002–2023). A) Annual publication volume of studies on Mechanical phenomena, B) Annual publication volume of studies on Research scale, C) Annual publication volume of studies on Scientific questions, D) Total publication volume in Plant Biomechanics by different countries.

The results of the spatiotemporal distribution analysis indicate that over the past two decades, the number of publications on mechanical research in plant science has shown a continuous upward trend. From a regional perspective, researchers in Europe, Asia, and North America made the most significant contributions, accounting for 38.3%, 31.8%, and 20.5% of all papers, respectively (Table S1, Supporting Information). At the country level, China, the United States, and Germany ranked in the top three in terms of publication volume, accounting for 19.4%, 12.8%, and 8.4% of the total, respectively (**Figure** **2**D). Research on mechanical phenomena showed a consistent upward trend from 2002 to 2023. Among these studies, “Stress and Strain” dominated the field with the highest publication output (29.3%), followed by “Structural Stability” (21.3%) and “Growth Mechanics” (20.9%). Notably, while “Fluid Mechanics” and “Viscoelasticity” received relatively less attention initially, they demonstrated substantial growth in recent years, particularly after 2019.

From the research scale perspective (Figure 2B), studies involving organ and microscopic scales dominated the field, accounting for ≈69.5% of total publications. Specifically, tissue‐scale research was the most prevalent (21.8%), followed by cellular‐scale (18.3%), and organ‐scale (17.9%) studies. Although subcellular‐scale research had a relatively lower total volume (11.7%), it showed significant growth in 2023. Research at macroscopic scales was comparatively less common, with limited publications at individual (9.1%) and population (4.0%) levels, mainly due to the technical challenges in characterizing large‐scale biomechanical phenomena. This distribution pattern not only reflects the current research priorities in plant biomechanics but also indicates potential directions for future breakthroughs.

Analysis of scientific questions reveals that “Mechanical Properties” and “Structure‐Function Relationships” dominated the field, contributing 30.9% and 28.2% of total publications respectively, followed by “Growth Mechanics” at 15.3%. Notably, while traditional research areas maintained steady growth, emerging topics such as “Biomimetic Applications” and “Ecological Mechanics” showed accelerated development, particularly after 2019. The year 2023 marked a significant shift, with “Structure‐Function Relationships” (277 papers) surpassing “Mechanical Properties” (245 papers) for the first time. This evolving pattern reflects an increasingly sophisticated approach in plant biomechanics research, characterized by enhanced integration across different scales and disciplines, suggesting a transition from isolated mechanical analyses toward a more comprehensive, multiscale, and interdisciplinary research paradigm.

## 3 The Evolution of Plant Biomechanics: A Paradigm Shift from Description to Systems Integration

**Figure 3:**
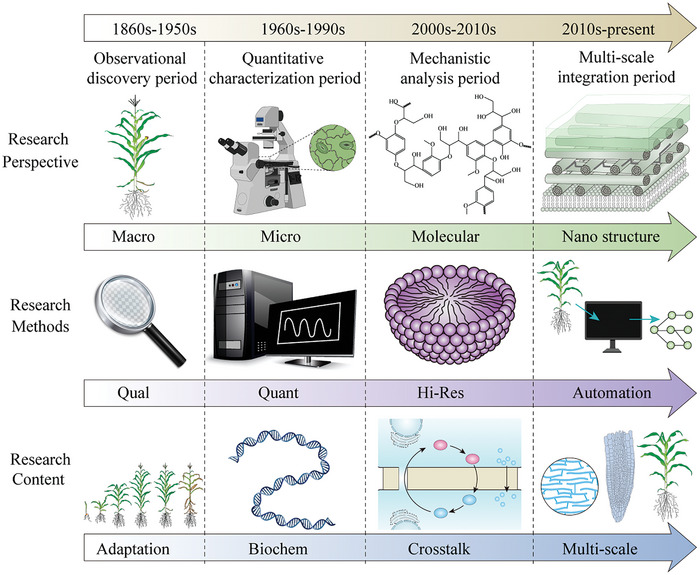
The developmental timeline of biomechanics in plant science research.

Plant biomechanics research has evolved through four major periods (**Figure** **3**): the observational discovery period (1860s–1950s), quantitative characterization period (1960s–1990s), mechanistic analysis period (2000s–2010s), and multi‐scale integration period (2010s‐present).

The descriptive mechanics phase was characterized by holistic observation and qualitative research. Knight (1806)^[^ ^9^ ^]^ demonstrated gravity's influence on plant growth through horizontal and vertical centrifugal experimental systems. This work established gravity as an external factor affecting plant organ directional growth. Darwin and his son (1883)^[^ ^10^ ^]^ revealed plants' sensitivity to mechanical stimuli through coiling experiments. Their work provided the first evidence of plants' ability to perceive and respond to mechanical signals. Research during this period relied primarily on visual observation and simple instruments. Mechanical analysis remained at a qualitative level, using descriptions such as “increased hardness” and “greater degree of bending”. Despite these limitations, this era established the phenomenological foundation for plant biomechanics.

The transition to the quantitative period marked significant advances in both research methodology and content. The introduction of specialized mechanical testing equipment enabled precise measurements of mechanical parameters. These methods included standardized tensile tests, which measure material strength by applying opposing forces until failure; indentation tests, which assess local mechanical properties through controlled probe penetration; and three‐point bending tests, which evaluate flexural properties by supporting a specimen at two points and applying force at a third point. Creep tests were also developed to measure time‐dependent deformation under constant load, providing crucial insights into cell wall viscoelastic properties. In theoretical modeling, approaches evolved from simple mathematical descriptions to more sophisticated frameworks. Continuum mechanics models treated plant tissues as continuous materials with averaged properties, while finite element modeling divided complex structures into smaller elements for detailed mechanical analysis. Coarse‐grained models bridged molecular and continuum scales by simplifying molecular interactions while maintaining essential mechanical behaviors.^[^ ^11^ ^]^ Research content expanded significantly during this period, with Cosgrove (1989)^[^ ^12^ ^]^ employing mechanical measurement techniques such as creep measurement, Instron tensile testing, and stress relaxation to reveal the crucial role of enzyme activity in plant cell wall extension. His subsequent work enriched the “acid growth theory” and expansion ‐mediated cell wall relaxation mechanisms.^[^ ^13^, ^14^ ^]^

The high‐resolution period marked a significant advance into microscopic‐scale research. New‐generation experimental techniques, represented by atomic force microscopy, optical tweezers, and micro‐indentation systems, enabled multi‐scale mechanical measurements from whole tissues to subcellular structures. These technological innovations allowed researchers to precisely quantify the physical properties of key structures such as cell walls, turgor pressure, and cytoskeleton, while opening new pathways for understanding the molecular mechanisms of plant cells' mechanical perception and response.^[^ ^15^ ^]^ Simulation methods also achieved breakthrough developments. Finite element analysis became capable of handling complex morphological changes, while molecular dynamics and coarse‐grained models pioneered new approaches for mechanical simulation of large‐scale molecular systems.^[^ ^15^, ^16^ ^]^ Research expanded to organ and tissue levels, with quantitative analyses revealing precise mathematical relationships between plant organs' mechanical properties and their internal structures. Notably, the spiral arrangement patterns of vascular tissues achieved optimal mechanical support with minimal material investment.^[^ ^17^, ^18^ ^]^ Hamant et al. (2008)^[^ ^19^ ^]^ discovered a mechanical feedback mechanism between tissue stress fields and microtubule cytoskeleton in Arabidopsis shoot apical meristem, providing the first evidence of how mechanical signals regulate plant tissue morphogenesis through the cytoskeleton. At the cellular level, studies revealed that cell wall mechanical properties undergo precise spatiotemporal regulation.^[^ ^6^ ^]^ The dynamic reorganization of the cytoskeleton not only determines individual cell growth direction but also coordinates tissue‐wide growth patterns through intercellular mechanical signal transmission.^[^ ^20^ ^]^

The current systems integration period demonstrates characteristics of multi‐scale and multi‐level research approaches. The mechanical transformation mechanisms from molecular to tissue levels have become a central focus. Coen and Cosgrove (2023)^[^ ^21^ ^]^ comprehensively elucidated the mechanical transmission mechanisms from cellulose microfibrils and cell walls to tissue levels during plant morphogenesis, revealing how plants achieve morphogenesis while maintaining mechanical strength. Methodologically, mechanical phenotype analysis has evolved toward greater precision and 3D characterization. Sasaki et al. (2023)^[^ ^22^ ^]^ exemplified this multi‐level research approach in their study of 3D cell wall structure formation in xylem vessels. They discovered that MAP70 proteins guide the directional growth of cell wall arch structures by regulating microtubule physical properties, simultaneously ensuring mechanical strength and maintaining water transport function in vessels. This finding not only revealed how plant cells construct precise 3D structures but also demonstrated the connection between molecular mechanisms and functional adaptability. In this research context, the study by Sleboda et al. (2023)^[^ ^23^ ^]^ on Mimosa pudica pulvini employed multi‐scale approaches. Through scanning electron and confocal microscopy observations, they revealed structural features across multiple levels: from subcellular cellulose microfibril arrangements to cellular plasmodesmata morphology and tissue‐level epidermal cell organization. Through osmotic experiments, they demonstrated how these multi‐level structures cooperatively guide hydraulic deformation, converting cellular volume changes into organ‐scale movements.

In the field of plant biomechanics, several influential works have emerged. Notable monographs include “Plant Biomechanics: An Engineering Approach to Plant Form and Function”,^[^ ^24^ ^]^ which laid the foundation for this field, followed by “Plant Physics”,^[^ ^17^ ^]^ “Plant Biomechanics: From Structure to Function at Multiple Scales”,^[^ ^25^ ^]^ and the recent “Plant Cell Walls: Research Milestones and Conceptual Insights”.^[^ ^26^ ^]^ Additionally, Journal of Experimental Botany published two special issues focusing on plant biomechanics in 2013 (Vol 64, Issue 15) and 2019 (Vol 70, Issue 14). These publications not only document the significant milestones in plant biomechanics research but also reflect its evolution from classical mechanical approaches to modern interdisciplinary perspectives, providing valuable insights into the historical development and future directions of this field.

## 4 Multi‐Scale Analysis of Plant Biomechanics Mechanisms: A Case Study on Maize Lodging Resistance

Maize stalk lodging represents a characteristic example of multi‐scale biomechanical processes. External wind forces induce bending deformation in plant stems, and lodging occurs when the bending moment exceeds the stem's flexural strength. The lodging resistance of maize is closely associated with the mechanical properties of its stems, which are dependent on structural characteristics across multiple scales from molecular to population levels, as well as plant‐environment interactions. This study examines maize lodging to illustrate research advances and methodologies in plant biomechanics across different scales, while identifying potential future breakthroughs. We examine maize lodging resistance from molecular to population scales, analyzing how structural features at each level contribute to overall mechanical stability (**Table** **1**).

**Table 1:** Multiscale biomechanical research framework for maize lodging.

Research Scale	3D Morphology Methods	Morphology Parameters	Mechanical Analysis Methods	Mechanisms
Molecular	X‐ray crystallography; Nuclear magnetic resonance spectroscopy; Single‐molecule force spectroscopy with AFM; Molecular dynamics simulation imaging	3D structure of biomacromolecules; Intermolecular forces and bonding patterns; Molecular conformation and spatial arrangement; Molecular chain length and orientation	Quantum mechanical calculations; Molecular dynamics simulation; Coarse‐grained simulation; Molecular force field parameterization; Molecular dynamics simulation of cell wall components	Structure‐property relationships at molecular level; Impact of intermolecular interactions on mechanical properties; Environmental factors (temperature, pH) affecting molecular conformation and mechanics; Energy transfer and dissipation mechanisms at molecular scale
Subcellular	Transmission electron microscopy; Cryo‐electron microscopy; Super‐resolution fluorescence microscopy; Electron tomography	Organelle morphology and spatial distribution; Cytoskeleton network structure; Membrane system organization; Internal transport channels	Cytoskeleton dynamics simulation; Membrane deformation mechanics; Stress‐strain analysis of subcellular components; Organelle interaction modeling	Relationship between cytoskeleton assembly and cellular mechanical stability; Impact of membrane system dynamics on mechanical response; Correlation between organelle spatial arrangement and mechanical signal transduction; Role of subcellular structures in mechanical stress adaptation
Cellular	Atomic force microscopy; Laser confocal microscopy; Micro‐computed tomography; Nano‐computed tomography	Cell wall thickness and layered structure; Cell wall nanomechanical properties (e.g., elastic modulus, hardness); Lignin and cellulose content and spatial distribution; Porosity and pore size distribution	Multiscale modeling of cell wall mechanical behavior (e.g., laminate model, micromechanical model); Finite element analysis of cell wall deformation and stress distribution; Three‐point bending tests for measuring mechanical properties	Correlation between cell wall chemical composition, nanostructure, and nanomechanical properties; Relationship between cell wall ultrastructure and compressive/bending resistance; Effect of cell wall composition and structure on crack initiation and propagation
Tissue/Organ	High‐resolution micro‐CT; Synchrotron X‐ray tomography; Serial block‐face scanning electron microscopy	3D spatial distribution and connectivity of vascular bundles; Vascular bundle size, shape, and wall thickness; Pith volume fraction and spatial arrangement; Tissue‐specific mechanical properties	Nonlinear finite element analysis of tissue/organ deformation and failure modes; Computational fluid dynamics analysis of sap flow in vascular bundles; Micromechanical modeling of tissue‐specific mechanical behavior; Multiscale structure‐function modeling of tissues and organs; Three‐point bending tests for measuring mechanical properties	Correlation between vascular bundle/pith 3D architecture and stem bending/compressive strength; Synergistic effects of vascular bundles and parenchyma tissues on mechanical properties; Influence of tissue heterogeneity and anisotropy on stress distribution and failure behavior; Multiscale mechanics of tissues and organs under different loading conditions
Individual organism	Terrestrial laser scanning; Airborne light detection and ranging; High‐resolution 3D imaging (e.g., structured light scanning, stereovision)	Leaf angle distribution and variation during growth; Internode length, diameter, and taper; Ear height and orientation; Root system architecture and anchorage strength; Biomass allocation pattern among organs	Finite element analysis of plant structural responses to wind and gravity loads; Computational fluid dynamics modeling of plant‐wind interactions; Functional‐structural plant modeling integrating morphology, biomechanics, and physiology; Multiscale modeling of plant lodging behavior; Three‐point bending tests for measuring mechanical properties	Relationship between plant 3D architecture, biomass allocation, and lodging resistance; Influence of organ morphology and mechanical properties on whole‐plant lodging behavior; Adaptive significance of plant architectural traits for wind and lodging resistance; Multiscale mechanical modeling of plant‐wind‐soil interactions and lodging processes
Population	UAV‐based laser scanning and photogrammetry; Airborne hyperspectral and thermal imaging; Satellite remote sensing (e.g., stereo imaging)	3D canopy structure and roughness parameters; Leaf area index (LAI) and vertical distribution; Canopy density and porosity; Canopy temperature and moisture distribution; Intra‐population variation and spatial heterogeneity	Large‐eddy simulation of canopy flow and turbulence structures; Computational fluid dynamics modeling of canopy‐wind interactions; Canopy radiative transfer and energy balance modeling; Coupling of canopy structure, microclimate, and crop growth models	Influence of canopy 3D structure on wind attenuation and turbulence generation; Relationship between canopy architectural traits and population lodging resistance; Canopy structural effects on light interception, temperature distribution, and photosynthesis; Multiscale modeling of canopy‐atmosphere interactions and their implications for lodging and crop productivity

### 4.1 Mechanical Properties and Regulatory Mechanisms of Plant Cell Wall Molecular Components

The primary plant cell wall comprises a complex network system of polysaccharide components at the molecular level, including cellulose, hemicellulose, and pectin.^[^ ^27^, ^28^ ^]^ These components determine the mechanical properties of cell walls through specific structural features and intermolecular interactions. Studies have demonstrated that genetic mutations affecting these components can lead to mechanical defects in plant organ development, subsequently impacting normal plant growth.^[^ ^29^ ^]^

Comparative analysis between wild‐type and cell wall mutants in Arabidopsis revealed distinct patterns of wall mechanics. AFM measurements showed that wild‐type plants maintain higher cell wall stiffness in slowly growing regions compared to rapidly elongating zones. Mutations affecting cellulose synthase or xylan biosynthesis result in reduced wall stiffness, while increased pectin methylesterification enhances wall rigidity through calcium‐mediated cross‐linking, thereby modulating growth rates.^[^ ^29^, ^30^ ^]^ These molecular‐level changes affect both individual cells and overall tissue mechanical properties and morphological development. In maize stalks, lodging resistance correlates strongly with cell wall component structures. The H‐lignin subunit content in secondary cell walls shows positive correlation with cortical penetration strength. Ferulic acid content positively correlates with lodging resistance, while diferulic acid content shows negative correlation.^[^ ^31^ ^]^ This indicates that stem strength and lodging susceptibility primarily depend on cell wall structural characteristics rather than absolute component content.

Molecular dynamics simulation has become an essential tool for studying plant cell wall molecular interactions and mechanical properties.^[^ ^32^, ^33^ ^]^ This computational approach enables nanoscale analysis of cellulose microfibril (CMF) ultrastructure and cellulose nanocrystal (CNC) construction characteristics through various force field systems (such as CHARMM and GLYCAM) and software packages (GROMACS and AMBER).^[^ ^34^, ^35^ ^]^ To enhance simulation reliability, researchers combine these methods with solid‐state nuclear magnetic resonance (ssNMR) and quantum mechanical calculations,^[^ ^36^ ^]^ while developing coarse‐grained molecular dynamics to facilitate integration between computation and experimentation. Despite technical limitations in simulating complex components, these methods provide crucial theoretical support for studying plant cell wall mechanical properties and reveal their structural characteristics and biosynthetic mechanisms at the nanoscale.

### 4.2 Physical Mechanisms and Mechanical Responses of Plant Cell Wall Growth

Cosgrove (2024)^[^ ^20^ ^]^ extensively summarized cell wall growth mechanisms from physical, cellular biological, and molecular signal transduction perspectives. The physical perspective established a comprehensive research framework from experimental observations to theoretical modeling, providing a fundamental basis for understanding cell wall growth. In physical models, growing cell walls are described as thin polymer shells stretched by turgor pressure. This growth process is primarily achieved through two key mechanisms: wall relaxation and stress relaxation drive water uptake by reducing turgor pressure, while polymer yielding and elastic contraction collectively lead to cell volume increase. Cell walls must also withstand mechanical stresses from various sources, including local stress variations due to cell morphology and large‐scale mechanical stresses from plant weight and tissue organ growth.^[^ ^13^ ^]^ At the experimental observation level, researchers mainly employ two methods: using constant‐force extensometers to measure irreversible cell wall extension (wall creep), or observing wall yielding through stress relaxation measurements.^[^ ^12^, ^37^, ^38^ ^]^ Recent computational models using molecular dynamics simulations have revolutionarily discovered that tensile forces are primarily transmitted directly through cellulose networks, rather than through traditionally assumed xylan connections or pectin network mediation.^[^ ^28^, ^39^ ^]^

These physical characteristics have significant applications in plant lodging resistance traits. Taking maize as an example, lodging‐resistant lines (such as B73) exhibit high rigidity and low plasticity, primarily achieved through maintaining stable lignin content and increasing strongly cross‐linked hemicellulose proportions. In contrast, lodging‐susceptible lines (such as EA2024) demonstrate high plasticity and low mechanical strength, associated with their higher cellulose content and flexible cell wall component distribution. These findings indicate that the degree of cross‐linking between cell wall components, particularly hemicellulose cross‐linking status, has a decisive impact on cell wall mechanical strength.^[^ ^40^ ^]^

### 4.3 Coupling Mechanisms Between Cellular Turgor Pressure Dynamics and Growth Regulation

Plant biomechanical research at the cellular scale primarily focuses on the relationship between turgor pressure and growth.^[^ ^41^ ^]^ Although traditional views considered turgor pressure as a passive driving force for cell wall expansion, recent studies reveal their relationship to be far more complex than anticipated.^[^ ^42^ ^]^ Current research describes this process through two theoretical frameworks: First, the Lockhart model, which elucidates the coupling relationships among osmosis, hydraulic conductivity, and cell wall elasticity, revealing that growth rate and turgor pressure are regulated by the balance of osmotic pressure, cell wall yield threshold, extensibility, and conductivity.^[^ ^43^ ^]^ Second, the Young‐Laplace equation, which describes the relationship among turgor pressure, tensile stress, and geometric shape in spherical cells, indicating that cell size and shape significantly influence growth dynamics.^[^ ^44^, ^45^ ^]^ Methodologically, this field combines theoretical modeling, experimental measurements of osmotic and turgor pressure, and analysis of cell wall mechanical properties. These studies reveal two important findings: turgor pressure can exhibit spatially heterogeneous distribution among different cells within a tissue and can be actively regulated;^[^ ^46^ ^]^ cell growth results from complex interactions among osmotic regulation, cell wall remodeling, and mechanical signal transduction, rather than being solely passively driven by turgor pressure.^[^ ^47^, ^48^ ^]^

This complex cellular‐level regulatory mechanism is also reflected in plant stem mechanical properties. As a fundamental mechanical factor maintaining plant tissue structural integrity, turgor pressure maintains stem mechanical strength by exerting pressure on cell walls,^[^ ^17^ ^]^ with its diurnal variations causing periodic fluctuations in stem mechanical properties.^[^ ^49^ ^]^ Simultaneously, as the driving force for cell growth, turgor pressure influences stem development and structural formation by regulating cell wall extensibility and remodeling processes.^[^ ^6^ ^]^ Under water stress conditions, decreased turgor pressure in primary tissues (particularly in parenchyma cells) reduces stem strength by compromising cellular rigidity and tissue mechanical properties, thereby increasing lodging risk. However, recent studies suggest that compared to stem morphological characteristics (such as basal density and stem diameter), which are mainly determined by secondary tissues with lignified cell walls, turgor pressure's direct contribution to lodging resistance through its effects on primary tissues is relatively minor.^[^ ^49^ ^]^ This finding provides a new perspective for understanding plant lodging resistance mechanisms, highlighting the dominant role of secondary tissue development in determining stem mechanical strength.

### 4.4 Mechanical Regulatory Networks in Plant Tissue and Organ Morphogenesis

At tissue and organ scales, plant biomechanical research primarily focuses on tissue mechanical properties (including elasticity, stiffness, and strength),^[^ ^17^, ^18^ ^]^ mechanical responses during growth (such as stress distribution and mechanical stimulation effects), ^[^ ^50^ ^]^ structural adaptability (such as morphogenesis and structural remodeling),^[^ ^26^ ^]^ and mechanical properties related to water transport (such as vascular system characteristics and cavitation phenomena).^[^ ^51^ ^]^ Research at tissue and organ scales primarily employs two types of observation equipment and modeling methods. For observation equipment, high‐resolution confocal microscopy is commonly used to observe vascular bundle arrangement and xylem vessel structural characteristics;^[^ ^52^ ^]^ X‐ray CT and MRI can non‐destructively reconstruct 3D structures of leaves, stems, and other organs,^[^ ^53^ ^]^ particularly crucial in studying vascular bundle distribution patterns;^[^ ^54^ ^]^ atomic force microscopy provides high‐resolution measurements of mechanical properties at the cellular level, allowing characterization of mechanical differences between adjacent tissue layers in root tip growth regions.^[^ ^55^ ^]^ Cellular force microscopy (CFM) combines the versatility of classical microindentation with high automation and resolution approaching that of atomic force microscopy, enabling large‐scale tissue mechanical property mapping and precise cell wall puncture experiments.^[^ ^56^ ^]^ For mechanical testing, researchers use material testing machines to study whole‐organ mechanical responses, such as evaluating lodging resistance of grass stems through tensile tests or analyzing dynamic changes in tissue hardness during fruit ripening through compression tests.^[^ ^57^ ^]^ Microscope‐coupled tensile testing devices, such as the Automated Confocal Micro‐Extensometer (ACME), enable simultaneous measurement of tissue mechanical properties and observation of cellular responses, providing insights into plant tissue mechanics at multiple scales.^[^ ^58^ ^]^

### 4.5 Multi‐Level Response Mechanisms of Plant Individual Mechanical Adaptability

At the individual scale, plant biomechanical research focuses on how plants sense and respond to mechanical environments, and how these responses affect plant growth, development, morphogenesis, and yield formation, with particular attention to adaptive responses and regulatory networks under external forces such as wind loads, gravity, and mechanical stress.^[^ ^59^, ^60^ ^]^ Specifically, plant biomechanical research at the individual scale has established systematic research methods and theoretical models. Research methods primarily include using 3D laser scanning and image analysis to obtain plant architectural characteristics such as plant height, stem inclination angle, and canopy structure;^[^ ^61^ ^]^ utilizing mechanical loading test systems to evaluate whole‐plant lodging resistance;^[^ ^62^ ^]^ and employing high‐throughput phenotyping platforms for dynamic monitoring of plant growth and development.^[^ ^63^ ^]^ Physical mechanics models incorporating gravity moments, wind loads, and support strength have been developed.^[^ ^64^, ^65^ ^]^ These methods reveal that plant lodging resistance is synergistically influenced by multiple factors including morphological characteristics (plant height, stem diameter, root distribution), mechanical properties (stem strength, root anchoring force), and physiological status (nutrient levels, hormone balance),^[^ ^66^ ^]^ showing significant mechanical adaptability.^[^ ^67^ ^]^

### 4.6 Scale Integration and Regulation of Crop Population Mechanical Properties

At the population research scale, plant biomechanical studies primarily rely on multi‐level observation equipment and multidimensional modeling methods. Observation equipment mainly includes remote sensing monitoring systems such as drones equipped with multispectral/hyperspectral cameras, satellite remote sensing platforms, and LiDAR systems. These devices can efficiently obtain population‐level canopy structure parameters, biomass distribution, and lodging conditions over large areas, and track population dynamic changes through temporal monitoring.^[^ ^68^, ^69^ ^]^ Ground monitoring equipment includes automatic weather stations, plant canopy analyzers, and high‐definition imaging systems, enabling precise observation of local environmental factors (wind speed, rainfall, temperature), population structural characteristics (leaf area index, plant height, density), and physiological properties.^[^ ^70^, ^71^ ^]^

Regarding modeling methods, researchers have developed physical mechanics models including population dynamics models, fluid‐solid coupling models, and multi‐body system models. These models describe the coordinated movement patterns of plant populations under wind loads, plant‐airflow interaction mechanisms, and mechanical coupling effects between plants.^[^ ^65^, ^72^, ^73^ ^]^ Meanwhile, prediction and warning models have been constructed, including deep learning‐based machine learning models, environment factor‐based statistical models, and integrated models incorporating multi‐source data.^[^ ^74^, ^75^ ^]^ Through mining historical data patterns and key influencing factors, these models enable early warning and dynamic assessment of population lodging risks. The organic combination of these observation equipment and modeling methods has not only deepened systematic understanding of plant population mechanical behavior but also provided scientific basis for optimizing crop lodging‐resistant cultivation techniques and developing disaster prevention measures, significantly contributing to improving crop yield stability and agricultural production efficiency.

## 5 Challenges and Difficulties in Plant Biomechanics Research

**Figure 4:**
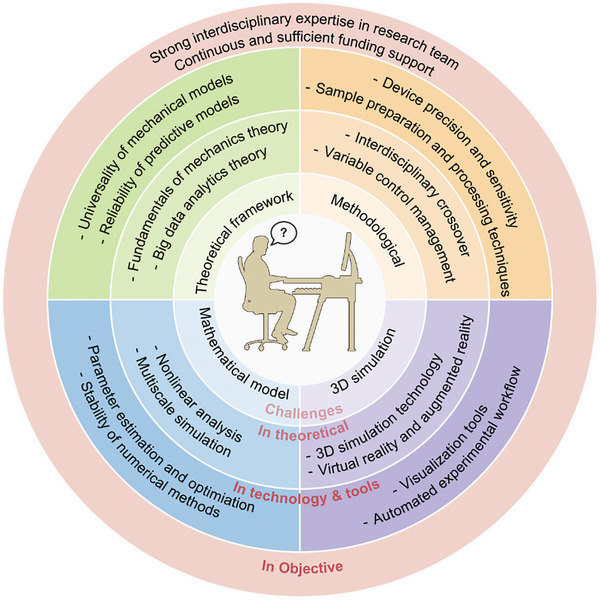
Challenges faced by biomechanics in plant science research.

In recent years, significant advancements have been achieved in plant biomechanics research, encompassing experimental methodologies, theoretical frameworks, and technical approaches. However, through systematic literature review and analysis of multi‐scale case studies, we have identified several crucial challenges in this field, primarily manifesting in research instrumentation and techniques, theoretical system construction, multi‐scale modeling, and structural characterization (**Figure** **4**). Addressing these challenges requires not only technological innovation and theoretical breakthroughs but also interdisciplinary integration and collaborative innovation.

### 5.1 Methodological Challenges: Scarcity of Advanced Equipment and Techniques

Current plant biomechanical research faces significant challenges in equipment, technology, and human resources. Advanced equipment requirements pose a major challenge. High‐precision instruments such as atomic force microscopes, optical tweezers, and micro‐indenters are expensive and complex to operate. These instruments require long‐term maintenance and calibration by specialized technicians. Many research institutions struggle with both the financial burden and the need for interdisciplinary technical teams.^[^ ^76^ ^]^ The inherent complexity of plant tissues and cellular structures creates unique measurement difficulties.^[^ ^77^ ^]^ Studying single‐cell mechanical properties requires precise isolation and measurement while maintaining cell viability and integrity. This demands sophisticated experimental techniques and specialized sample preparation methods with controlled environmental conditions.^[^ ^78^ ^]^ The multilayered structure of plant cell walls, mechanical connections between cells, and tissue heterogeneity further complicate accurate mechanical measurements. The interdisciplinary nature of plant biomechanics requires expertise across multiple fields. Researchers must combine knowledge from biology, physics, materials science, and engineering. They need skills in plant physiology, mechanical analysis, mathematical modeling, and computer programming. This broad skill requirement often creates barriers to entry for new researchers.^[^ ^79^ ^]^ Data analysis presents additional challenges. Modern biomechanical research generates high‐dimensional, multi‐scale, and nonlinear data. Processing requires advanced statistical methods, machine learning algorithms, and specialized software. Researchers must develop strong computational skills, which increases research complexity.^[^ ^80^ ^]^ Experimental reproducibility and reliability remain ongoing concerns. Individual plant variation, environmental fluctuations, and measurement uncertainties can affect result accuracy. The lack of standardized methods and evaluation systems makes it difficult to compare and validate different studies.^[^ ^6^ ^]^

### 5.2 Lack of Theoretical Framework: Cognitive Dilemma in Multi‐Scale Mechanical Properties

Plant biomechanics currently lacks a comprehensive theoretical framework that can fully explain and predict plant mechanical properties.^[^ ^81^ ^]^ The factors affecting plant cellular and tissue mechanics are highly complex. These include cell wall biochemical composition, microstructure, turgor pressure, cell division, growth, environmental conditions, and physiological states.^[^ ^82^ ^]^ The dynamic coupling and interaction of these factors across multiple scales pose significant challenges for theoretical modeling.^[^ ^83^ ^]^ Current theoretical systems have notable limitations. Most models can only describe single‐scale phenomena or specific events, making cross‐scale theoretical integration difficult. For example, a unified mathematical framework for mechanical property transmission from molecular to cellular to tissue levels remains elusive. The quantitative relationships between cell wall multicomponent structure and its macroscopic mechanical properties, along with their dynamic regulation during plant growth and development, require deeper theoretical explanations. While experimental methods can measure and observe plant mechanical properties, translating these phenomenological insights into quantitative mechanistic explanations and predictions remains challenging.^[^ ^84^ ^]^ This requires researchers to have not only solid biological knowledge but also deep understanding of physics, mathematics, and statistics.^[^ ^85^ ^]^ Key challenges include the integration of multidisciplinary theoretical approaches, limitations in computational model accuracy and applicability, and coordination between experimental design and theoretical predictions. Rapid advances in experimental techniques have provided unprecedented detailed data, but theoretical research has lagged behind.^[^ ^86^ ^]^ Major challenges include systematic interpretation of high‐throughput experimental data, bridging gaps between model predictions and experimental validation, and converting qualitative understanding into quantitative descriptions. Developing effective multi‐scale theoretical models to explain experimental results and elucidate cross‐scale mechanical behavior and biological mechanisms remains a crucial task in plant biomechanics research.^[^ ^87^ ^]^

### 5.3 Modeling Bottlenecks: Severe Challenges in Multi‐Scale Dynamic Simulations

Current mathematical models face numerous challenges in characterizing plant mechanical properties, particularly in capturing the dynamic complexity of plant life processes.^[^ ^88^ ^]^ Models must simultaneously account for structural complexity and diversity of plants, as well as multi‐level effects of environmental factors such as temperature, humidity, and light on plant growth.^[^ ^89^ ^]^ Plant biomechanics research spans multiple scales, from molecular‐level cell wall structures to whole‐plant morphology and movement. This vast spatial and temporal range presents unprecedented modeling challenges.^[^ ^90^ ^]^

Traditional mathematical tools, including partial differential equations, nonlinear dynamics, and fractal theory, prove inadequate in describing complex plant dynamic processes. This limitation has driven researchers to explore and develop new mathematical theories and methods.^[^ ^16^ ^]^ Model developers must possess solid mathematical foundations and programming skills to understand and implement these complex mathematical tools. Such interdisciplinary capabilities are rare in traditional plant biology laboratories.^[^ ^91^ ^]^

Computational resource requirements pose additional challenges. Model development, solution, and optimization often involve processing massive datasets, requiring high‐performance computing platforms.^[^ ^92^ ^]^ Particularly in handling complex mechanical problems, researchers may need to develop new numerical algorithms or use specialized software packages, further raising technical barriers (Figure 4). Model simplifications and assumptions require rigorous validation, as inappropriate simplifications can lead to significant deviations in model accuracy and predictive power.^[^ ^93^ ^]^ For example, oversimplified assumptions about material homogeneity or neglecting environmental effects on local mechanical properties can result in substantial errors in estimating key mechanical parameters during plant dynamic behavior simulation.

### 5.4 Technical Shortcomings: Lack of 3D Structural Characterization of Plants

Traditional 2D models oversimplify plant structural complexity, failing to accurately simulate the spatiotemporal distribution of mechanical behaviors such as stem torsion and bending, leaf curling, and complex root network dynamics.^[^ ^94^ ^]^ 2D models cannot fully describe complex behaviors like spiral growth and torsional deformation in plant stems, accurately express multi‐directional stress distributions during leaf curling, or comprehensively characterize root system spatial distribution and mechanical responses.

While 3D reconstruction techniques based on 2D slice data partially address these limitations, they still suffer from information loss and resolution constraints, making it difficult to fully characterize complex mechanical interactions between plant structures and their environment.^[^ ^95^ ^]^ This approach particularly struggles with continuous dynamic processes, often losing critical intermediate state information. 3D imaging and modeling techniques can fundamentally overcome 2D study limitations by precisely characterizing plant structural configurations and their mechanical regulatory mechanisms across cellular, tissue, and organ scales.^[^ ^96^ ^]^

However, 3D structural characterization and simulation demand sophisticated experimental and computational capabilities, requiring specialized 3D imaging equipment and complex image processing and reconstruction algorithms (Figure 4). The plant science field currently has limited technical expertise in these areas.^[^ ^97^ ^]^ Existing imaging technologies struggle to achieve both wide‐field view and high resolution simultaneously, with insufficient capability to capture rapid biological processes in real‐time. Live sample non‐invasive observation techniques need improvement, and deep tissue observation remains limited by optical transparency. Selective labeling methods for specific structures require enhancement, and integration of complementary imaging techniques needs strengthening.

Data processing presents additional challenges. Massive 3D image data storage and management create substantial pressure, while noise reduction and image enhancement algorithms require optimization. Technical bottlenecks exist in automatic recognition and segmentation of complex biological structures, and innovative methods are needed for multi‐dimensional data statistical analysis and feature quantification. Meeting plant 3D structural characterization demands requires developing high‐resolution, multimodal, multi‐scale 3D imaging techniques and establishing rapid, accurate specialized 3D reconstruction methods.^[^ ^98^ ^]^ This necessitates collaborative innovation among plant scientists, engineers, and computer scientists.^[^ ^99^ ^]^

## 6 Future Research Prospects

### 6.1 Advancing Plant Biomechanics through Artificial Intelligence

**Figure 5:**
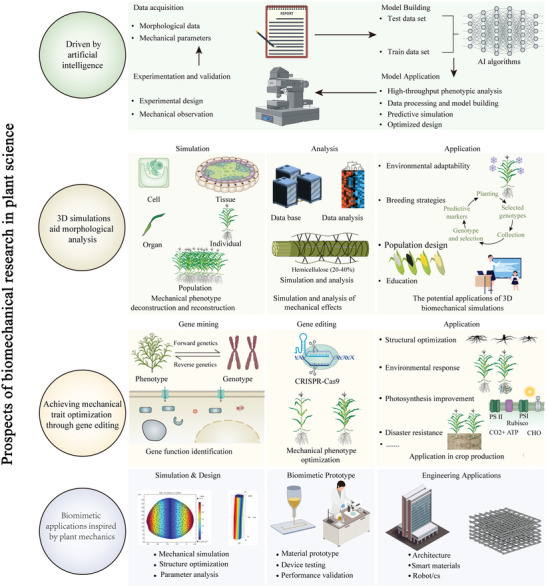
Prospects of plant biomechanics research.

Future research in plant biomechanics will likely be propelled by artificial intelligence (AI) technologies, particularly deep learning algorithms (**Figure** **5**). These advanced tools can efficiently analyze vast amounts of plant image data, differentiate subtle phenotypic traits, and track dynamic growth processes. AI's ability to mine complex, multidimensional datasets will facilitate the construction of sophisticated mathematical models simulating plant biomechanical properties. Recent breakthroughs exemplify this potential, such as Microsoft Research Asia's AI^2^BMD system,^[^ ^100^ ^]^ which achieves first‐principles level accuracy in simulating biomolecular dynamics. This system can efficiently model structures containing over 10000 atoms at full atomic resolution, operating several orders of magnitude faster than traditional quantum mechanics methods like density functional theory (DFT). By employing specialized protein fragmentation methods and creating datasets of 20 million snapshots, such AI‐driven systems demonstrate how machine learning can revolutionize molecular modeling and simulation. Such models will not only enhance our understanding of existing data but also predict plant performance under novel environmental conditions (Figure 5). The success of AI^2^BMD in exploring previously undetectable protein conformational spaces suggests similar potential for plant biomechanics, where AI could reveal hidden mechanical properties and dynamic behaviors. This predictive capacity will enable researchers to simulate plant behavior under various growth stages and stresses, evaluate breeding strategies and agronomic practices, optimize designs, and inspire new biomimetic materials. Ultimately, the integration of AI in plant biomechanics research promises to accelerate discoveries, potentially revolutionizing sustainable agriculture and materials science (Figure 5). As demonstrated by AI^2^BMD's achievements in molecular dynamics, AI‐driven approaches can bridge the gap between computational efficiency and accuracy, opening new possibilities for understanding complex biological systems at multiple scales.

### 6.2 Multi‐Scale 3D Morphological Simulation: Decoding Plant Structural Complexity

Multi‐scale 3D morphological simulation approaches plants as dynamic, multi‐layered systems. By integrating multimodal imaging techniques with advanced computational models, this method enables analysis of plant structure's 3D morphology and mechanical properties across scales, from microscopic cells to entire plants (Figure 5). This approach elucidates how biomechanics influences morphological phenomena at various levels. Beyond static analysis, the technology employs time‐series analysis and multi‐physics field simulations to reveal plants' mechanical responses to dynamic forces such as wind, animal activity, and water impact. This allows for the prediction of critical conditions that may lead to structural damage or failure. The multi‐scale 3D morphological simulation not only assesses existing varieties' performance under extreme environments but also tests the effects of gene editing or breeding strategies in virtual space (Figure 5). Consequently, it guides morphological and functional optimization, facilitating the development of new plant lines with enhanced stress resistance and high productivity. Additionally, this approach provides a foundation for precision agronomic practices (Figure 5).

### 6.3 Integration of Biomechanics, Genetic Analysis, and Gene Editing: A Comprehensive, Precise Strategy for Optimizing Plant Production Performance

The integration of biomechanics research with genetic analysis and gene editing technologies forms a comprehensive strategy for optimizing plant performance. This approach constructs a cross‐scale systematic framework that elucidates how plant biomechanical properties are driven by complex networks of gene‐environment interactions (Figure 5). Based on this deep understanding, precise regulatory tools such as gene editing can be employed to directly modify key genes influencing plants' physical properties and biomechanical functions. This enables optimization of multiple traits including stress resistance, growth habits, and yield, which holds significant application value in agricultural production and related industries. The strategy not only aims to enhance existing traits but also, leveraging new technologies like gene editing, can endow plants with novel characteristics and functions to adapt to specific environments. This approach propels fundamental research forward, deepening our understanding of plant complexity and opening new research perspectives and directions (Figure 5).

### 6.4 Biomimetic Applications Inspired by Plant Mechanics

Biomimetic applications based on plant biomechanics demonstrate immense potential for future development. For instance, leaf structures alone have inspired innovations across multiple engineering disciplines, ranging from mechanical and civil engineering to energy and chemical engineering.^[^ ^101^ ^]^ These applications stem from leaves' intrinsic properties including photosynthesis efficiency, stress sensing, self‐cleaning abilities, and various mechanical adaptations (Figure 5). Pioneering work by Speck et al. has demonstrated several successful biomimetic applications, including self‐healing elastomers inspired by latex‐bearing plants, adaptive attachment systems based on climbing plants, and innovative façade shading systems inspired by the Bird‐of‐Paradise flower. Their research has shown how plant mechanical principles can be effectively translated into technical solutions with high reliability and sustainability. Researchers can utilize computational simulation technologies to analyze the mechanical properties of plant structures and mechanisms in depth, develop innovative engineering design solutions, and transform these concepts into practical technologies through prototype validation.^[^ ^102^, ^103^ ^]^ The interdisciplinary nature of this field is evident in how a single biological structure like a leaf can inform solutions in fluid dynamics, optics, electronics, and materials science. Future breakthroughs in this field may emerge in areas such as self‐healing materials, morphing structures, efficient water transport systems, and environment‐responsive architecture (Figure 5). As bioinspired engineering principles become increasingly integrated with modern technologies, plant biomechanics research will provide novel approaches to solving engineering challenges and drive the implementation of more sustainable innovative solutions. This interdisciplinary research approach not only promotes the advancement of fundamental plant biomechanics research but also promises to deliver a series of revolutionary engineering applications, making significant contributions to the sustainable development of human society (Figure 5). The comprehensive integration of advanced structures and functional materials inspired by leaf designs exemplifies how biological principles can be translated into practical engineering solutions across diverse technological domains.

## 7 Conclusion

Plant biomechanics research has undergone a remarkable transformation, evolving from traditional qualitative observations to sophisticated quantitative analyses across multiple scales. This review highlights the field's crucial role in understanding plant structure‐function relationships and its growing importance in modern agriculture and ecological conservation. Through systematic examination of research developments, particularly exemplified by maize lodging studies, we have identified both significant progress and persistent challenges in this field. The integration of classical mechanical principles with cutting‐edge biological techniques has opened new avenues for investigating plant phenotypes. However, several key challenges remain: 1) the need for more robust theoretical frameworks that can bridge multiple spatial scales, 2) the development of advanced methodologies for precise mechanical measurements at cellular and tissue levels, and 3) the refinement of 3D simulation models that can generate meaningful predictions of plant responses to mechanical stresses, thereby guiding researchers to explore underlying mechanisms and novel biological phenomena. Looking forward, we anticipate that emerging technologies will revolutionize plant biomechanics research. The application of artificial intelligence and machine learning algorithms will enhance our ability to process and interpret complex biomechanical data. Multi‐scale modeling approaches will better integrate molecular, cellular, and organismal levels of analysis. Advanced genetic tools will enable more precise manipulation of mechanical properties in crops. Additionally, biomimetic applications inspired by plant mechanical adaptations may lead to innovative solutions in agricultural engineering and environmental protection. These developments position plant biomechanics at the forefront of addressing critical challenges in sustainable agriculture and ecosystem resilience. As this field continues to mature, its insights will become increasingly valuable for crop improvement programs, cultivation practices, and ecological conservation strategies. The future of plant biomechanics lies in its ability to combine theoretical innovation with practical applications, ultimately contributing to global food security and environmental sustainability.

## Conflict of Interest

The authors declare no conflict of interest.

## Supporting information



Supplemental Table 1
